# Quantitative evaluation of fiber fuse initiation with exposure to arc discharge provided by a fusion splicer

**DOI:** 10.1038/srep25366

**Published:** 2016-05-03

**Authors:** Shin-ichi Todoroki

**Affiliations:** 1 National Institute for Materials Science, Namiki 1-1, Tsukuba, Ibaraki 305-0044, Japan

## Abstract

The optical communication industry and power-over-fiber applications face a dilemma as a result of the expanding demand of light power delivery and the potential risks of high-power light manipulation including the fiber fuse phenomenon, a continuous destruction of the fiber core pumped by the propagating light and triggered by a heat-induced strong absorption of silica glass. However, we have limited knowledge on its initiation process in the viewpoint of energy flow in the reactive area. Therefore, the conditions required for a fiber fuse initiation in standard single-mode fibers were determined quantitatively, namely the power of a 1480 nm fiber laser and the arc discharge intensity provided by a fusion splicer for one second as an outer heat source. Systematic investigation on the energy flow balance between these energy sources revealed that the initiation process consists of two steps; the generation of a precursor at the heated spot and the transition to a stable fiber fuse. The latter step needs a certain degree of heat accumulation at the core where waveguide deformation is ongoing competitively. This method is useful for comparing the tolerance to fiber fuse initiation among various fibers with a fixed energy amount that was not noticed before.

Heat-induced strong absorption of silica glass optical fiber at more than 1050 °C^1^ plays a potential risk in many fiber optics applications including optical communication and “power over fiber”, as a form of fiber fuse phenomenon^2^,^3^,^4^,^5^. In the 28 years since its discovery, many techniques have been used to initiate a fiber fuse, namely local heating of a fiber segment. However, the process of fiber fuse initiation has only been investigated in two papers^6^,^7^ whose findings will be summarized later. By contrast, many researchers in optical communication have determined that the threshold power for fiber fuse propagation, *P*_th_, is, for example, less than 1.5 W for SMF-28(e) fibers^3^,^8^,^9^,^10^,^11^, which is widely regarded as the maximum allowable power if we are to avoid the problem. In fact, this parameter tells us nothing about fiber fuse initiation because the initiation must occur only after the accumulated energy has exceeded a certain value in a small area. Thus, we need additional information to enable us to discuss this phenomenon, such as the accumulation time and the heat flow in the area. Here I review previous studies from this viewpoint.

In 1995, Davis *et al.* investigated the initiation behavior for various types of fibers pumped with 1.06 *μ*m light^6^. They stated that “the most reliable way to initiate was to contact the end of the fiber with a metallic object”. The minimum initiation power, *P*_init_, ranged from 0.40 W to 4.0 W, but “at lower power levels, initiation might take several attempts over minutes”. In 2002, Yanagi *et al.* reported that a fiber fuse was initiated between two uncleaned surfaces of MU-type fiber optic connectors after more than 160 mating cycles^7^. The cable was dispersion-shifted fiber (DSF) pumped with 1.48 *μ*m light at 2 W but there was no description of the irradiation time and the absorber was not identified. These two results clearly show certain limited possibilities but we cannot reproduce them or generalize the requirements for the initiation.

To establish a reproducible initiation method with a fixed irradiation time, I performed a fiber fuse initiation experiment at the interface between a fiber end and a commercially available highly Co-doped borosilicate glass^12^. A stable fiber fuse was initiated by the five-second irradiation of 5.5 W laser light at 1480 nm through an SMF-28e+ fiber where the Co ions converted the light power to heat. However, this evaluation method requires many trials to obtain reliable initiation probability values because molten borosilicate glass is insufficiently rigid to maintain a stable reaction area.

Here I propose a new geometry with an arc discharge provided by a commercially available fusion splicer, where the initiation occurs only in silica glass. In addition, the light absorption of the waveguide is measurable over time, and the external heating intensity is variable since it is independent of the pump laser power. This improvement helps us to initiate a fiber fuse with higher reproducibility and to reveal the process on the basis of the correlation between the injected energy and the deformation modes of the fibers.

## Results

**Figure 1:**
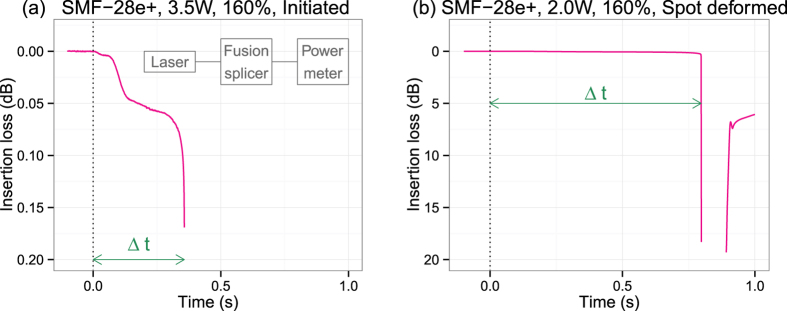
Decay curves of insertion loss observed during (**a**) fiber fuse initiation and (**b**) spot deformation. Photographs of these samples are seen at the top of Fig. 3(c) and in the middle of Fig. 3(a), respectively.

Two single-mode silica glass optical fibers, that were one of the four types listed in Table 1, were fusion-spliced to form an optical connection between a Raman fiber laser (PYL-10-1480, IPG Laser, 1.48 *μ*m) and an optical power meter (see the inset in Fig. 1(a)). Before the fiber was removed from the splicer, laser light at a certain power was injected into the fiber, and an arc discharge was applied for one second at a point 1 mm from the splice point directed toward the laser. This positional shift is needed to minimize the heat-induced modification caused by the prior splicing at the fiber segment for the initiation test. The time-varying insertion loss was monitored to determine the decay time, Δ*t*, from the onset to the fall as shown in Fig. 1. This loss was caused by the deformation of the waveguide structure and the heat-induced absorption of the glass. The damage sites were observed with a digital optical microscope.

**Figure 2:**
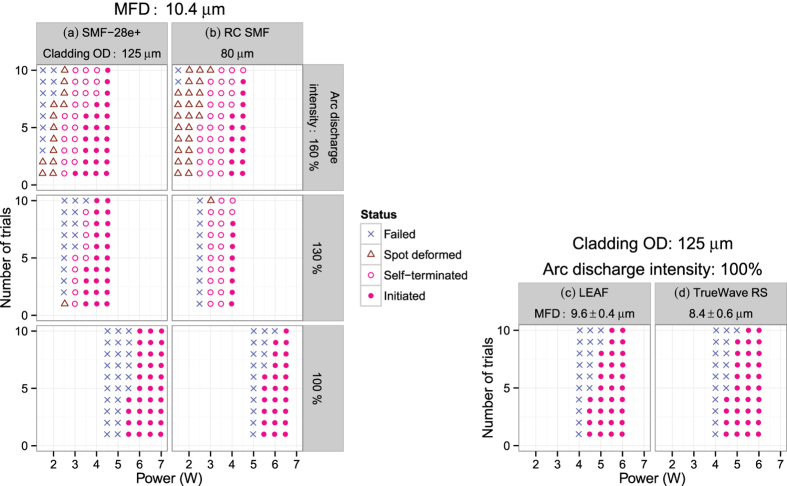
Histogram of status of heated segment (see Fig. 3) Fiber: (**a**) SMF-28e+, (**b**) RC SMF, (**c**) LEAF, and (**d**) TrueWave RS. Laser: 1480 nm.

The heat needed to initiate a fiber fuse in this system is provided by the arc discharge and the pump laser. The laser light cannot heat the fiber at room temperature but is converted to heat at an elevated temperature through partially deoxidized SiO_2_^13^. When the arc discharge intensity was the same as that for normal fiber splicing (denoted below as “100%”), the minimum laser power for initiation was 5.5 W for SMF-28e+. This was determined by a series of initiation tests where the laser power was changed in increments of 0.5 W, and ten trials were performed at each laser power. There were two possible results, namely initiated or failed without any visible deformation in the fiber, as shown at the bottom of Fig. 2(a). All the trials pumped at more than 6 W resulted in an initiation.

**Figure 3:**
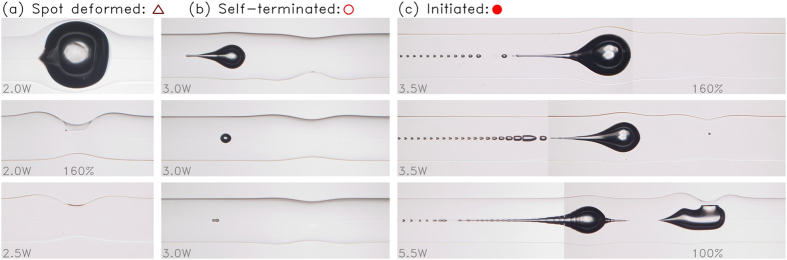
Photographs of deformed areas classified as (**a**) spot-deformed, (**b**) self-terminated, and (**c**) initiated (see text). Arc discharge power is 140% unless otherwise specified.

It is reasonable to expect that the minimum power for initiation will be reduced as the arc discharge intensity increases. However, the actual situation was more complicated. Namely, new failure modes appeared between the two types; self-terminated after initiation and failed with spot deformation. All the samples were classified into one of these four types based on their micrographs, some of which are shown in Fig. 3.

Concerning the “spot deformed” samples, the insertion loss sometimes recovered as shown in Fig. 1(b). This recovery is due to a bubble bursting and the subsequent reformation of a void-free glass bridge. In all the self-terminated samples, no periodic void train appeared in the core region. This means that the reaction region was extinguished before reaching a steady state of fiber fuse propagation.

**Figure 4:**
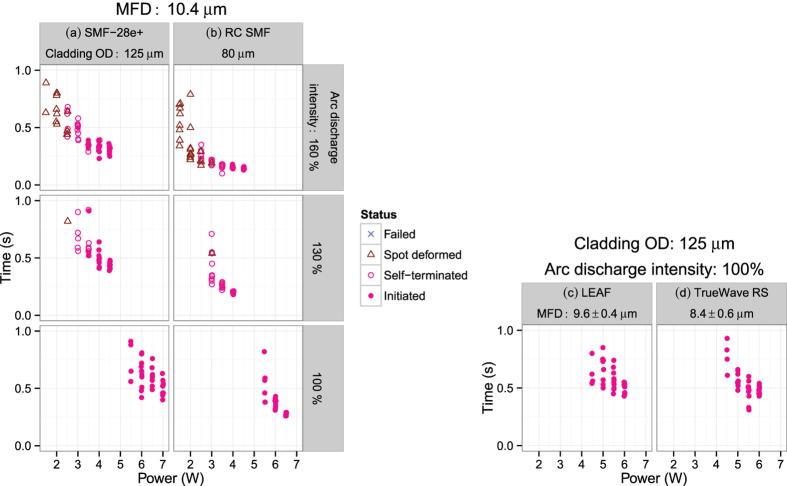
Decay time of insertion loss, Δ*t* (see Fig. 1). Fiber: (**a**) SMF-28e+, (**b**) RC SMF, (**c**) LEAF, and (**d**) TrueWave RS. Laser: 1480 nm.

Figure 2(a) maps this classification for three arc discharge intensities, 100%, 130% and 160% for SMF-28e+. As the discharge intensity increased, the maximum laser power causing no modification decreased while the minimum power for initiation remained at around 3.5 W. A further increment in the discharge intensity to 200% pumped at 1.5 W caused the whole fiber segment to melt. Figure 4(a) maps the corresponding decay time, Δ*t*, which decreased as the laser power and discharge intensity increased.

Similar behavior was observed for the RC SMF samples as shown in Figs 2(b) and 4(b) except for the following two points. (1) Melting occurred at a discharge intensity of 180% pumped at 1.5 W. This value is smaller than that for SMF-28e. (2) The minimum Δ*t* value is smaller than those for SMF-28e+ for each discharge intensity. These two fibers have the same mode field diameter (MFD) but RC SMF has a smaller cladding outer diameter as listed in Table 1. Therefore, these observations are due to the RC SMF’s smaller cladding diameter, namely, the shorter time for heat penetration to the core.

On the other hand, the minimum initiation laser powers for LEAF and TrueWave RS were 4.5 W as shown in Fig. 2(c,d). This value is 1 W smaller than that for the other two fibers. This shift is also seen in the Δ*t*-power plot shown in Fig. 4. These results are caused by an increment in power density due to their smaller MFD.

## Discussion

**Figure 5:**
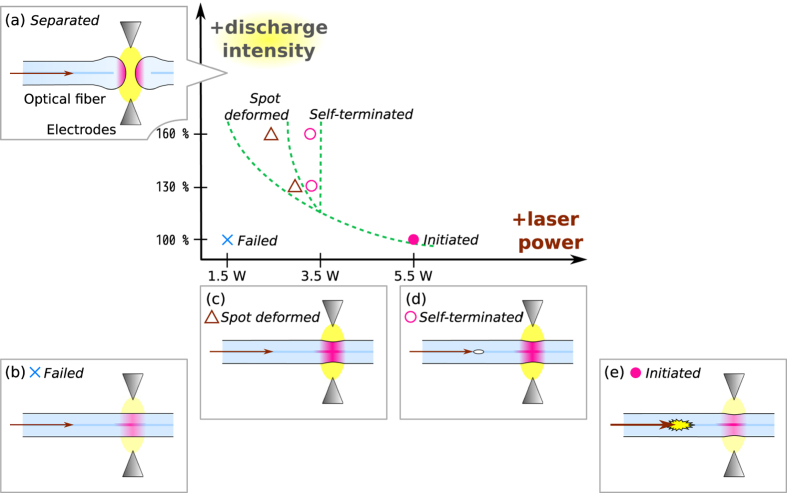
Summary of the initiation test with illustrations of the experimental configuration and deformation geometry.

Consequently, four failure modes were found in these initiation tests for SMF-28e+ and RC SMF, and these modes are mapped in Fig. 5 in a qualitative plot of arc discharge intensity and laser power along with illustrations showing the geometry of the system. This correlation provides a clue for understanding the initiation process, i.e., the heat flow balance between the laser and the external heating.

The laser light is converted to heat only once the temperature of the fiber core has exceeded 1050 °C^1^,^3^. This secondary heat flow forms a hot spot at the fiber core. When the arc discharge intensity is 100%, a laser power of 5.5 W is the border between initiation and no modification (see Fig. 5(b,e)). This means that this is the minimum power needed to heat the core region and initiate a fiber fuse with one second of arc discharge exposure.

This border power does not simply decrease as the arc discharge intensity increases because the hot spot size expands due to the reduction of the heat flow from the core and the enhancement of the external heat flow. This expansion becomes a hurdle as regards initiating a fiber fuse through a small fiber core. This is why spot deformed samples appeared with an arc discharge intensity of more than 100% (see Fig. 5(c)).

There is another failure mode, the self-terminated mode, as shown in Fig. 5(d), where the reaction region was extinguished after it moved away from the hot spot. It appears because the laser power is insufficient to make the reaction region grow into a steady state of a fiber fuse running through a cold waveguide. (Similar structure was reported in a heated end of a cleaved fiber^14^.) The border between the self-terminated and initiated modes is about 3.5 W (SMF-28e+) and 4 W (RC SMF) for arc discharge intensities of both 130% and 160%. This is because the termination point is away from the center of the hot spot. Thus, the border power depends solely on the fiber dimensions. Namely, the border for RC SMF is larger than for other fibers because the thinner cladding results in much grater inner heat dissipation.

In summary, the presence of these two failure modes shows that the fiber fuse initiation process consists of two steps, i. e., the generation of a precursor in a hot spot and the transition to a steady state of fiber fuse propagation out of the hot spot. With an arc discharge intensity of 100%, once the first step has started, the second step occurs successively because the hot spot is sufficiently small. It generally takes less time to complete these two steps (Δ*t*) as the laser power and discharge intensity increase as shown in Fig. 4.

The minimum laser power in this study was 1.5 W, which is close to but larger than the *P*_th_ of the four fibers^3^. However, no fiber fuse initiation was observed with this pump power even if the arc discharge intensity was increased to the value at which the fiber melted (see Fig. 5(a)). Therefore, the fiber fuse initiation requires not only sufficient laser energy but also a sufficient concentration of energy into the fiber core. A certain degree of energy concentration is needed to generate a precursor (the first step discussed above) and a certain amount of laser power exceeding *P*_th_ is necessary to make it grow into a fiber fuse out of the hot spot (the second step).

This mechanism helps us to understand our empirical facts on fiber fuse initiation, that is, the lower the laser energy is, the longer we have to continue the trial for initiation. A precursor appears at a moment when an energy accumulation at the core exceeds a certain value before waveguide deformation. Its frequency depends on the energy flow mode at the heated core region; namely, the waveguide geometry (fiber end/segment, cladding thickness, hole configuration, etc.), heat generation source (external heat, light-heat conversion center including partially deoxidized SiO_2_), and accumulation time. In addition, heat-induced modification of the wavewuide structure also changes the energy flow mode and the reaction area.

In short, the precursor of fiber fuse appears in a non-equilibrium system where heat accumulation and waveguide deformation are ongoing competitively. Thus, the present experimental setup gives us a highly reliable method with a quantitative energy flow into the reaction area in the viewpoint of time and space. It is highly reliable because we can find (1) the experimental conditions required for “initiation without fail” in at least ten trials, and (2) an apparent relationship between Δ*t* and given energy. Moreover, the following two qualitative relations are confirmed with an arc discharge intensity of 100%. (1) Δ*t* depends on the thickness of the cladding. (2) the minimum laser power for initiation depends on the MFD. Therefore, this method is expected to be a feasible way to test the tolerance to fiber fuse initiation and fiber deformation of various modern fibers including multi-core fibers and few-mode fibers^15^,^16^.

As for other initiation techniques that have been reported so far, their geometry is not appropriate for controlling the energy amount quantitatively; namely, blocking light with wooden object, cutting by a wire stripper^14^, and applying white correction fluid at the tip of fiber^8^, etc. In these cases, it can be possible to determine the minimum initiation power, *P*_init_, if the examiner has a patience to continue the trials over minutes. The resulting value is merely a record and hard to be compared fairly with others because of the lack of reproducibility.

## Methods

### Fusion splicing

The additional arc discharge intensity denoted as 100% for all four fibers was the same in the present fusion splicer, Furukawa Electric, S183PM.

## Additional Information

**How to cite this article**: Todoroki, S. Quantitative evaluation of fiber fuse initiation with exposure to arc discharge provided by a fusion splicer. *Sci. Rep.* **6**, 25366; doi: 10.1038/srep25366 (2016).

## Figures and Tables

**Table 1:** Optical fibers tested in this study and their specifications; cladding outer diameter (OD) and mode field diameter (MFD).

Fiber	Supplier	Cladding OD (*μ*m)	MFD @ 1550 nm (*μ*m)
(a) SMF-28e+	Corning	125	10.4 ± 0.5
(b) RC SMF	Corning	80	10.4 ± 0.8
(c) LEAF	Corning	125	9.6 ± 0.4
(d) TrueWave RS	OFS	125	8.4 ± 0.6

## References

[b1] Kashyap R. Self-propelled self-focusing damage in optical fibres. In Duarte F. J. (ed.) *Lasers’87; Proc. the 10th Int. Conf. Lasers and Applications*, 859–866 (STS Press, McLean, VA, 1988). (Lake Tahoe, Nevada, USA, Dec. 7–11, 1987).

[b2] Kashyap R. & Blow K. J. Observation of catastrophic self-propelled self-focusing in optical fibres. Electron. Lett. 24, 47–49 (1988).

[b3] Todoroki S. Fiber fuse—light-induced continuous breakdown of silica glass optical finer. NIMS Monographs (Springer: Japan, Tokyo,, 2014). 10.1007/978-4-431-54577-4

[b4] Stone W. C., Hogan B., Siegel V., Lelievre S. & Flesher C. Progress towards an optically powered cryobot. Annals of Glaciology 55, 1–13 (2014).

[b5] Audo F. *et al.* Study of the optical phenomena in a 10 km long optically powered data link dedicated to a submarine cabled observatory. In *Proceedings of 2013 MTS/IEEE OCEANS*, 1–9 (IEEE, 2013). (Bergen, Norway, 10–13 June 2013). 10.1109/OCEANS-Bergen.2013.6608174.

[b6] Davis D. D., Mettler S. C. & DiGiovani D. J. Experimental data on the fiber fuse. In Bennett H. E., Guenther A. H., Kozlowski M. R., Newnam B. E. & Soileau M. J. (eds.) *27th Annual Boulder Damage Symposium: Laser-Induced Damage in Optical Materials: 1995*, vol. 2714 of *SPIE Proceedings*, 202–210 (SPIE, 1996). (Boulder, CO, USA, 30 Oct. 1995). 10.1117/12.240382.

[b7] Yanagi S., Asakawa S. & Naruse R. Characteristics of fiber-optic connector at high-power optical incidence. Electron. Lett. 38, 977–978 (2002).

[b8] Abedin K. S., Nakazawa M. & Miyazaki T. Backreflected radiation due to a propagating fiber fuse. Optics Express 17, 6525–6531 (2009). 19365477 10.1364/oe.17.006525

[b9] Dianov E. M. *et al.* Catastrophic destruction of optical fibres of various composition caused by laser radiation. Quantum Electron. 32, 476–478 (2002).

[b10] Rocha A. M., Domingues F., Facão M. & André P. S. Threshold power of fiber fuse effect for different types of optical fiber. In *the 13th International Conference on Transparent Optical Networks (ICTON 2011)*, 1457–1549 (IEEE, 2011). (Tu.P.13; Stockholm, Sweden, 26–30 June 2011). 10.1109/ICTON.2011.5971025.

[b11] Todoroki S. Threshold power reduction of fiber fuse propagation through a white tight-buffered single-mode optical fiber. IEICE Electronics Express 8, 1978–1982 (2011).

[b12] Todoroki S. Quantitative evaluation of fiber fuse initiation probability in typical single-mode fibers. In *Optical Fiber Communication Conference* (Optical Society of America, 2015). (W2A.33; Los Angeles, CA, 25 March 2015). 10.1364/OFC.2015.W2A.33.

[b13] Shuto Y., Yanagi S., Asakawa S., Kobayashi M. & Nagase R. Fiber fuse phenomenon in step-index single-mode optical fibers. IEEE J. Quantum Electronics 40, 1113–1121 (2004).

[b14] Atkins R. M., Simpkins P. G. & Yablon A. D. Track of a fiber fuse: a Rayleigh instability in optical waveguides. Opt. Lett. 28, 974–976 (2003). 12836750 10.1364/ol.28.000974

[b15] Richardson D. J., Fini J. M. & Nelson L. E. Space-division multiplexing in optical fibres. Nature Photonics 7, 354–362 (2013).

[b16] Saitoh K. Multicore fiber technology. In *Optical Fiber Communication Conference* (Optical Society of America, 2015). (Th4C.1; Los Angeles, CA, 26 March 2015). 10.1364/OFC.2015.Th4C.1.

